# Comparing the Diagnostic Accuracy of Bedside Lung Ultrasonography and High-Resolution Computed Tomography of the Thorax in Acute Chest Trauma Patients: A Study Protocol

**DOI:** 10.7759/cureus.78292

**Published:** 2025-01-31

**Authors:** Krishna C Gongati, Aditya Pundkar, Charuta Gadkari

**Affiliations:** 1 Department of Emergency Medicine, Jawaharlal Nehru Medical College, Datta Meghe Institute of Higher Education and Research, Wardha, IND; 2 Department of Orthopedics, Jawaharlal Nehru Medical College, Datta Meghe Institute of Higher Education and Research, Wardha, IND; 3 Department of Anesthesia, Jawaharlal Nehru Medical College, Datta Meghe Institute of Higher Education and Research, Wardha, IND

**Keywords:** acute chest trauma, hrct thorax, lung ultrasonography (lus), pneumothorax, real-time imaging

## Abstract

Background

Emergency rooms frequently see cases of chest trauma, which require prompt and precise assessment to determine the best course of action. Traditional diagnostic techniques like CT scans and chest X-rays have restrictions related to radiation exposure, cost, and mobility. Due to its mobility, radiation-free nature, and real-time imaging capabilities, bedside lung ultrasonography (LUS) has become a potential modality for assessing chest injuries. The main goal is to evaluate the accuracy of bedside LUS and high-resolution computed tomography (HRCT) thorax in patients with acute chest trauma.

Materials and methods

A bedside LUS will be performed on 45 adult patients over the age of 18 who present to the emergency department with severe chest trauma. An HRCT thorax will be done at the radiodiagnosis division. To evaluate LUS's diagnostic accuracy in identifying different traumatic conditions such as pneumothorax, hemothorax, and pulmonary contusion, its results will be compared with those of HRCT thorax.

Results

After the study is completed in 2025, conclusions will be made.

Conclusions

The accuracy of bedside LUS in diagnosing traumatic lung injury will be compared to HRCT thorax findings, and conclusions will be drawn.

## Introduction

Blunt chest trauma continues to be a significant global source of morbidity and death, posing a considerable challenge to emergency room physicians. point-of-care ultrasonography (POCUS) has emerged as the go-to screening technique in many emergency departments throughout the world for suspected bodily trauma [1]. The best use for it is extended focused abdominal sonography for trauma (EFAST). It is essential to assess blunt chest trauma injuries precisely and quickly to provide the best possible care and outcomes for the patient [1]. Bedside ultrasonography has several potential advantages over traditional diagnostic modalities, including immediate availability, portability, non-ionizing radiation, and real-time dynamic imaging capability [2]. These advantages make ultrasonography a preferred diagnostic method for patients with chest injuries in the acute setting, when time-sensitive diagnosis is essential [2].

In contrast to computed tomography (CT), the current study examines the possible clinical use of chest ultrasonography to identify lung contusions, pneumothorax, and hemothorax in the emergency department. The identification of minor anomalies is improved by the real-time monitoring of dynamic processes made available by ultrasonography, such as lung expansion and pleural slip [3].

Despite these hopeful qualities, the usefulness of bedside ultrasonography in the initial evaluation of patients with severe chest injuries is still being investigated and discussed. Studies have shown that ultrasonography can identify some types of chest injuries; nevertheless, there are still issues with its accuracy, consistency, and limits compared to more conventional imaging modalities like CT scanning [4]. The first evaluation of patients with catastrophic chest injuries using bedside ultrasonography is still being studied and discussed. Although studies show that ultrasonography may help diagnose some types of chest injuries, concerns remain over its accuracy, consistency, and limits compared to more conventional imaging modalities such as CT scanning [5].

Imaging technologies are essential for managing critically sick patients because they improve the effectiveness of therapeutic and diagnostic therapies. The utility of bedside chest radiography and thoracic CT is limited by a number of variables [6]. This study evaluates the diagnostic accuracy of bedside ultrasonography in a sample of patients who have experienced severe chest injuries to contribute to the growing body of evidence in favor of using ultrasonography in the early diagnosis and management of trauma patients [7,8]. In summary, the investigation of bedside ultrasonography as a primary adjunct in the assessment of chest trauma patients represents a critical area of research with significant implications for clinical practice. Through rigorous examination and analysis, this thesis endeavors to elucidate the role of ultrasonography in enhancing the diagnostic capabilities of healthcare providers and improving outcomes for patients with traumatic chest injuries.

## Materials and methods

The research makes use of a prospective observational design with patients who arrive at the emergency room with chest trauma. The study begins after clearance from the institutional ethics committee and informed consent from participating patients. Each patient receives a thorough clinical evaluation consisting of a comprehensive medical history and a physical exam focusing on the thoracic region. To evaluate patients, healthcare providers employ the ABCDE (airway, breathing, circulation, disability, exposure) method, a structured approach for promptly assessing and managing critically sick or injured individuals. Subsequently, for patients with multiple traumas or suspected abdominal injuries, lung ultrasonography (LUS) utilizing the EFAST protocol is conducted to evaluate the abdomen for fluid collections and organ damage. Following this, a radiologist performs transthoracic ultrasonography within four hours of chest trauma using a device equipped with a high-resolution curvilinear linear transducer (3.5-5 MHz). The preferred patient position for this procedure is sitting with the arm on the affected side raised to or above the head, which helps widen the intercostal spaces. However, for patients in critical condition, the examination is carried out with the patient lying flat or in a semi-recumbent position at a 45-degree angle. A non-contrast chest CT serves as the benchmark for ultrasonography comparison. The CT examination uses a 32-multislice CT machine in the radiology unit. The scan parameters are as follows: helical acquisition mode, 120 kV voltage, 25 mA current, 1.25 mm helical slice thickness, 1 mm interval, and a field of view of 351, scanning from the renal arteries to the neck root. Patients with ongoing or worsening symptoms undergo additional monitoring and evaluation. The findings of LUS are compared with those of conventional imaging to assess its diagnostic accuracy in detecting various traumatic pathologies such as pneumothorax, hemothorax, and pulmonary contusion.

Inclusion and exclusion criteria

The adult population of any gender who sustains chest trauma in a civilian scenario and is transferred to the emergency medicine department of Acharya Vinoba Bhave Rural Hospital within four hours of chest trauma is included in the study. Patients with penetrating injuries are excluded. Patients who are not willing to participate in the study are excluded. Post-cardiopulmonary resuscitation patients are excluded. Patients who are intubated after the trauma are excluded from the study.

Statistical analysis

The sample size calculation is done using Slovin's formula:

\[ n=N1+Nd2n = \frac{N}{1 + Nd^{2}}n=1+Nd2N​ \]

Considering the adult population of any gender who sustains chest trauma in a civilian scenario and is transferred to the emergency medicine department of Acharya Vinoba Bhave Rural Hospital, N = 50, d = estimated error = 5%.

\[ n=501+50(0.05)2=45n = \frac{50}{1 + 50(0.05)^{2}} = 45n=1+50(0.05)250​=45 \]

The minimum sample size required is 45.

Descriptive statistics, including average, standard deviation, median, and range (minimum to maximum), are used to present numerical data. For categorical information, summaries comprise frequencies and percentages. A chi-squared (χ²) test examines associations between categorical variables. The analysis includes the computation of crucial diagnostic parameters such as sensitivity, specificity, and positive and negative predictive value. A p-value less than 0.05 is considered statistically significant.

Ethical approval

Approval is sought from the Institutional Ethics Committee of Datta Meghe Institute of Higher Education and Research under approval no. DMIHER(DU)/IEC/2024/39 and registered in the Clinical Trials Registry of India (CTRI) under no. CTRI/2024/08/071868. Written informed consent is obtained from all patients included in the study.

## Results

Data is compiled in an Excel sheet (Microsoft Corporation, Redmond, WA, USA) and analyzed using appropriate statistical tests. The results will be drawn in 2025 after the study is completed. The study aims to evaluate whether bedside LUS combined with high-resolution computed tomography (HRCT) thorax could be essential in managing acute chest trauma patients, potentially enhancing patient safety and clinical outcomes.

## Discussion

The evaluation of patients with acute chest trauma relies on rapid and accurate diagnosis of thoracic injuries. HRCT and bedside LUS are two commonly utilized imaging modalities [9]. While HRCT is considered the gold standard for thoracic injuries, LUS has emerged as an accessible and efficient tool in emergency settings. A detailed comparison of their accuracy, particularly in trauma cases, reveals the strengths and limitations of each modality [10].

LUS has gained prominence in emergency care due to its bedside accessibility and non-invasive nature. The effectiveness of ultrasonography protocols, such as the BLUE (Bedside Lung Ultrasonography in Emergency) protocol, facilitates rapid assessment of thoracic conditions, including pneumothorax, pleural effusion, and lung contusions [1]. LUS significantly improves decision-making in acute trauma cases, offering results comparable to traditional imaging methods, especially when time is a critical factor [8].

LUS exhibits excellent sensitivity and specificity in trauma situations when pneumothorax and hemothorax are frequent occurrences. LUS performed by emergency physicians has been found to be extremely accurate in identifying traumatic pneumothorax following a brief training session, highlighting its usefulness in situations where time is of the essence [11]. The importance of LUS in locating the "lung point," a pneumothorax-specific signal, improves diagnostic precision [12].

Additionally, LUS is used to diagnose rib fractures, as demonstrated by Battle et al., who found that LUS is more sensitive than chest radiography in detecting rib fractures. The utility of LUS extends beyond trauma in diagnosing pneumonia and pleural effusions, conditions that may accompany thoracic trauma [3].

HRCT remains the gold standard for diagnosing thoracic trauma. HRCT provides detailed visualization of the lung parenchyma, pleura, airways, and thoracic wall, allowing for a comprehensive assessment of injuries such as pulmonary contusions, rib fractures, and hemothorax. Its superiority over other imaging techniques, including chest X-rays and even LUS, lies in its ability to detect more minor and subtle injuries. HRCT is unmatched in its sensitivity for detecting blunt traumatic injuries [13].

While HRCT provides superior anatomical detail, it requires transportation to the radiology suite and exposes patients to ionizing radiation. In critical trauma cases, delays due to patient transport can be detrimental [14]. The need for rapid diagnosis in unstable trauma patients thus limits the applicability of HRCT as a first-line diagnostic tool [15].

Studies comparing the two modalities show that bedside LUS is more successful in quickly identifying potentially fatal conditions such as pleural effusion, pulmonary contusions, and pneumothorax. Bedside LUS reveals pleural effusion as an anechoic or hypoechoic space between the pleura. Pulmonary contusions appear as irregular, hypoechoic areas, often with B-lines due to alveolar damage. Pneumothorax is identified by the absence of lung sliding, the "barcode sign," and the absence of B-lines on LUS [3,12,14]. LUS has demonstrated sensitivity comparable to CT in detecting lung contusions in trauma patients [5,16]. In patients who have suffered blunt trauma, POCUS accurately identifies thoracoabdominal lesions. However, for more complex injuries or those not visible on ultrasonography, HRCT remains essential [2,17,18].

Early LUS is a valuable tool for early intervention because it can predict the onset of acute respiratory distress syndrome in trauma patients. However, HRCT is indispensable for the comprehensive evaluation of thoracic structures, particularly when subtle injuries are suspected [19].

Although LUS offers significant advantages, it has limitations. Its diagnostic accuracy depends heavily on operator experience, and specific injuries, such as deep parenchymal or small subpleural lesions, may be missed by LUS but detected on HRCT [15]. Furthermore, while ultrasonography is highly sensitive in identifying pneumothorax and pleural effusion, it may not fully replace HRCT in detecting fractures and more complex thoracic injuries [20].

Despite these limitations, LUS provides several benefits, especially in emergency or resource-limited settings, where its portability, rapidity, and safety make it an invaluable tool. Its non-invasiveness and absence of radiation also offer significant advantages over HRCT, particularly for repeat examinations or in populations vulnerable to radiation exposure, such as pregnant women or children [21].

## Conclusions

A conclusion will be drawn regarding the accuracy of bedside LUS in detecting various traumatic pathologies in acute chest trauma patients. The integration of LUS as a primary assessment adjuvant could revolutionize the approach to managing acute trauma patients in the ED. This research emphasizes the significance of ongoing innovation and assessment in medical practice to enhance patient care and results.
